# Diversity of Ticks in the Caribbean Region and Detection of Their Pathogens Using BioMark Technology

**DOI:** 10.1155/tbed/8135946

**Published:** 2025-12-05

**Authors:** Roxanne A. Charles, Emmanuel Albina, Mathilde Gondard, Rosalie Aprelon, Clemence Galon, Mark Trotman, Colbert Bowen, Sharmine Melville, Kofi Sylvester, Lisa Musai, Michael Morris, Christopher Oura, Sara Moutailler, Karla Georges

**Affiliations:** ^1^ Basic Veterinary Sciences, School of Veterinary Medicine, The University of the West Indies, Uriah Butler Highway, Mt. Hope, Trinidad and Tobago, uwi.edu; ^2^ CIRAD, UMR ASTRE, F-97170, Petit Bourg, Guadeloupe, France, cirad.fr; ^3^ ASTRE, University of Montpellier, CIRAD, INRAE, F-34398, Montpellier, France, cirad.fr; ^4^ UMR Virology, Animal Health Laboratory, ANSES, INRAE, National Veterinary School of Alfort, Maisons-Alfort, 94700, France, vet-alfort.fr; ^5^ UMR BIPAR, Animal Health Laboratory, ANSES, INRAE, National Veterinary School of Alfort, Maisons-Alfort, 94700, France, vet-alfort.fr; ^6^ Food and Nutritional Security, Ministry of Agriculture, St. Michael, The Pine, Barbados; ^7^ Ministry of Agriculture, Regent and Vlissengen Roads, Georgetown, Guyana; ^8^ Veterinary and Livestock Services Division, Ministry of Agriculture, Fisheries, Forestry, Food Security and Rural Development, Union, Castries, Saint Lucia; ^9^ Department of Agriculture, La Guerite, Basseterre, Saint Kitts and Nevis; ^10^ Animal Production and Health, Ministry of Agriculture, Land and Fisheries, #80 Abercomby Street, Port of Spain, Trinidad and Tobago

**Keywords:** Caribbean, cattle, dogs, microfluidic real-time PCR, tick-borne pathogens, ticks

## Abstract

**Introduction:**

Ticks and the pathogens they transmit are widespread in Caribbean animal populations. There is; however, limited information on the diversity of ticks and tick‐borne pathogens (TTBPs) in the region. This study aims to identify TTBPs across multiple Caribbean countries using a high‐throughput real‐time microfluidic PCR system.

**Methods:**

Six Caribbean territories: Barbados, Guyana, St. Kitts, St. Lucia, Tobago and Trinidad were targeted in this study. Nucleic acids were extracted from individual ticks, and a high‐throughput microfluidic real‐time PCR system was used to screen for 49 bacterial species (10 genera) and 18 protozoan species (six genera). Five tick species were detected using morphological or molecular techniques.

**Results:**

A total of 840 ticks were tested from 155 dogs and 111 cattle. Morphological and molecular diagnostic methods detected five tick species: *Rhipicephalus sanguineus* sensu lato (s.l.), *Rhipicephalus microplus*, *Amblyomma variegatum*, *Amblyomma ovale* and *Amblyomma cajennense* s.l. Overall, the DNA of 18 pathogens belonging to eight genera was detected in 22.5% (189/840) ticks obtained from both cattle (96/335) and dogs (93/505). The most prevalent pathogens were *Anaplasma marginale* 14% (47/335) and *Hepatozoon canis* 3.4% (17/505) in cattle and dog ticks, respectively. Dual and triple infections were also detected in 3.3% (*n* = 28) and 0.6 % (*n* = 5) of tested ticks, respectively. The DNA of *Ehrlichia ruminantium* was detected in *R. sanguineus* and *A*. *variegatum* from dogs in Barbados‐ a first record for this island. Another key finding was the novel detection of a *Borrelia* sp. in a *R. sanguineus* s.l. tick from Trinidad.

**Conclusion:**

The high diversity of pathogens detected in this study, with some being of veterinary and public health importance, highlights the strength of the high‐throughput microfluidic real‐time PCR system as a surveillance tool for the efficient and rapid detection of tick‐borne pathogens (TBPs) of veterinary, public health and economic significance in the Caribbean.

## 1. Introduction

Ticks and tick‐borne pathogens (TTBPs) cause significant morbidity and mortality in domestic animals and threaten public health globally [1–3]. For example, losses caused by infestation of cattle with *Rhipicephalus microplus* have been estimated to be USD$13.9–187 billion annually, worldwide [1]. Economic losses caused by ticks may be due to reduced hide quality, loss of condition, low productivity, the high costs of treating infested animals and their chemical control in the environment [4]. The archipelago of Caribbean islands is a Neotropical Zone located in the Western hemisphere, between North and Central America. This region is separated from the Pacific Ocean by Mexico and Central America to the west, bounded to the north by the Greater Antilles, to the east by the Lesser Antilles, and to the south by South America. Guyana is located on the northern coast of South America, bordered by the Atlantic Ocean to the north, Brazil to the south and southwest, Venezuela to the west and Suriname to the east.

The tropical landscape of the six territories sampled, varying from tropical rainforest to tropical savannahs, is favourable for the survival and dispersal of ticks and their pathogens. Additionally, the undocumented movement of people and animals across porous borders aids in the introduction of new tick vectors and tick‐borne pathogens (TBPs) [5, 6].

Ixodid ticks, including the tropical cattle tick, *Rhipicephalus* (*Boophilus*) *microplus*, the brown dog tick, *Rhipicephalus sanguineus* sensu lato (s.l.), tropical lineage, renamed *Rhipicephalus linnaei* [7] and the tropical bont tick (TBT), *Amblyomma variegatum*, are the main tick species of veterinary and medical importance in the Caribbean region [8–10]. However, other species such as *Amblyomma cajennense* s.l. (the Cayenne tick) and *Amblyomma ovale* have also been reported in the region, although they have been implicated in the transmission of fewer TBPs in animals and humans [6, 11–14].

*R. microplus* is a one‐host tick and economically the most important pest of cattle worldwide [15]. It is involved in transmitting *Anaplasma marginale*, *Babesia bigemina* and *Babesia bovis*, which cause bovine anaplasmosis and babesiosis, respectively. These notifiable pathogens are endemic in the Caribbean and are responsible for economic losses to the livestock industry [16]. This tick may also feed on other domestic animals in the Caribbean, such as buffaloes, sheep, goats, horses, donkeys, pigs, dogs, wildlife and humans [8, 17, 18].

*R. sanguineus* s.l. ‘tropical lineage’, recently renamed *R. linnaei* [7], is a three‐host tick (larval, nymphal and adult stages on different hosts) commonly found on dogs throughout the Caribbean and globally [3, 19]. This tick occasionally parasitises other hosts, including humans. It is a competent vector of many disease agents of veterinary and zoonotic importance, including *Babesia vogeli*, *Coxiella burnetii*, *Ehrlichia canis*, *Hepatozoon canis* and *Rickettsia* spp., which have been reported in the region [9, 20–23].

*A. variegatum*, commonly known as the TBT, is a three‐host tick found in several Caribbean territories. It parasitises a broad range of hosts with a particular preference for cattle [24, 25]. This species poses a significant constraint to ruminant production, serving as the primary vector of *Ehrlichia ruminantium*, the causative agent of heartwater, a notifiable and often fatal ruminant disease. Additionally, *A. variegatum* is associated with acute dermatophilosis in cattle, caused by the bacterium *Dermatophilus congolensis* [15, 25]. Other pathogens transmitted by this tick include *Rickettsia africae*, the causative agent of African tick‐bite fever in humans, as well as *Theileria mutans* and *Theileria velifera*, which affect cattle [26–30].

Reports on ticks (~56 tick species) and TBPs have been previously recorded in the Caribbean [9, 23, 30, 31]. However, current epidemiological data remain limited for many islands/territories, with previous studies focusing primarily on serological and molecular detection of pathogens in the mammalian host [9]. Notably, the most recently published data (2024) on bacterial and protozoan pathogens in ticks from the currently sampled territories is a single case study from Tobago [14]. Given the potential for the re‐emergence and persistence of tick‐borne diseases (TBDs) in the Caribbean, there is a clear need for enhanced surveillance and updated epidemiological insights into TTBPs. To address this gap, this present study employed a large‐scale, high‐throughput molecular screening tool that builds upon earlier work conducted in the French Caribbean islands [30]. This expanded approach incorporated a broader geographic range and a more comprehensive diagnostic panel utilising microfluidic real‐time PCR technology (BioMark dynamic array, Standard BioTools, USA). This system enables the simultaneous detection of up to 96 targets in 96 samples, performing up to 9216 individual, real‐time PCR reactions per run. Though relatively novel in the Caribbean, this technology has been previously applied in Europe, the French Antilles and Cuba for the detection of TTBPs in ixodid ticks [30, 32, 33]. This study, therefore, aims to provide critical new insights into the epidemiology of TTBPs in the Caribbean, contributing to improved surveillance and informing future strategies for controlling the occurrence and re‐emergence of TBDs in the region.

## 2. Materials and Methods

### 2.1. Collection of Epidemiological Data, Tick Collection and Identification

Ticks were collected from animals on farms (cattle and dogs) and households (dogs) in this study. This was part of the European Union (EU) funded DOMOTICK project in collaboration with the French Agricultural Research Centre for International Development (CIRAD), CaribVET, and the University of the West Indies, for the discovery of TBPs (bacteria, parasites, and viruses) in Caribbean ticks. Permission was granted by the Chief Veterinary Officers of all territories to handle animals for sampling, and consent was obtained from all farm animal and household owners before tick and data collection. This study was approved by the Ethics Committee of the Faculty of Medical Sciences, the University of the West Indies (Approval Number CEC206/05/16). Ticks (any species, sex, and life stage) were collected from cattle and dogs between December 2016 and 2019 from Barbados, Guyana, St. Kitts, St. Lucia, Tobago, and Trinidad. A maximum of 10 ticks were collected per animal and 30 ticks per location. Ticks were removed with thumb forceps and placed in labelled collection tubes (one tube per animal). Ticks were then transported alive at ambient temperatures from the field to the respective laboratory locations in the six Caribbean territories. At the laboratories, all live ticks were immediately frozen at −20°C to −80°C. Frozen ticks were packaged in insulated containers and transported via priority air couriers to Guadeloupe for DNA extraction. Before DNA extraction, all ticks were morphologically identified under a dissection microscope using established taxonomic identification keys and micrographs [34–36]. It should be noted that DNA extraction was not performed on all ticks collected in this study due to limited resources. Instead, a representative sample of at least three ticks (one female, male and nymph, if present) was used from at least one animal per farm.

### 2.2. DNA Extraction

DNA was extracted from individual ticks after washing in 1 mL of phosphate‐buffered saline (PBS 1X) for 2–3 min at 7 Hz/s in the TissueLyser (Qiagen, Hilden, Germany). A steel ball was used to crush frozen ticks with the TissueLyser, followed by an automated extraction using the Biomek4000 automated platform (Beckman Coulter, Villepinte, France). The total nucleic acid per tick sample was eluted in 160 µL of RNase‐Free Water and stored at −80°C for further analysis [30].

### 2.3. Primers and Probe Design

The list of pathogens and ticks (excluding *A. ovale*), their targeted genes, and the primers/probes utilised are available in Table S1. Some of the oligonucleotides were designed explicitly for the Caribbean study, while others were derived from a previous study [30, 32]. All primer/probe sets were validated for a range of dilutions of positive controls, including cultures, DNA samples, and plasmids, by real‐time TaqMan PCR assays on a LightCycler 480 (LC480; Roche Applied Science, Germany). Real‐time PCR assays were performed with a LC480 Probe Master Mix 1X (Roche Applied Science, Germany) with 200 nM primers and probes in a final volume of 12 and 2 µL of control DNA. Thermal cycling conditions were as follows: 1 cycle for 5 min at 95°C, 45 cycles at 95°C for 10 s and 60°C for 15 s, and one final cooling cycle at 40°C for 10 s.

### 2.4. Pre‐Amplification of DNA Samples

Enhancement of pathogen DNA was achieved by pre‐amplification using the PerfeCTa PreAmp SuperMix (Quanta Biosciences, Beverly, USA) according to the manufacturer’s instructions. All TBP primers were pooled with a final and equal concentration of 45 nM each. Pre‐amplification reactions were performed in a 5 µL mixture containing 1 µL of PerfeCTa PreAmp SuperMix (5X), 1.25 µL of pooled primer mix (forward and reverse), 1.25 µL of DNA, and 1.5 µL of MilliQ water. This reaction mixture was cycled once at 95°C for 2 min and 14 cycles at 95°C for 10 s and 60°C for 3 min. The reaction mixtures were diluted 1:10 at the end of the cycling programme [30]. Pre‐amplified DNA was stored at −20°C until further use.

### 2.5. Microfluidic Real‐Time PCR Assay

The BioMark real‐time PCR system (Standard BioTools Inc., formerly Fluidigm Corp, South San Francisco, CA, USA) was used for high‐throughput microfluidic, real‐time amplification. For this study, the 48.48 Dynamic Array IFC chips (Standard BioTools Inc., USA) were employed. Each chip allowed the analysis of 48 samples and 48 PCR mixtures to generate 2304 individual reactions. This assay is described in detail in two previous studies [30, 32].

Data was acquired using the BioMark Real‐Time PCR system, and threshold cycle (Ct) values were analysed with the Fluidigm Real‐Time PCR Analysis software. All field samples were analysed in duplicate. Three controls were used to validate the experiments on each chip. They included a negative water control to rule out contamination, a DNA extraction control corresponding to primers and probes targeting the portion of the 16S rRNA gene of ticks, and an internal control composed of DNA from *Escherichia coli* strain EDL933 targeting the *eae* gene to check for the presence of PCR inhibitors according to a previous study [30].

### 2.6. Confirmation of Results by PCR and Sequencing

Conventional/Nested PCR was done using primers targeting genes or regions distinct from those used by the BioMark system to confirm the presence of targeted DNA in both field samples and positive controls (Table 1). PCR products were sequenced using Sanger sequencing at Eurofins MWG Operon (Biomnis‐Eurofins Genomics, France). Sequences were assembled using the BioEdit software (Ibis Biosciences, Carlsbad, CA, USA). The online basic local alignment search tool (BLAST) search was used to compare nucleotide sequences found in this study with reference sequences published in the GenBank databases (NCBI).

**Table 1:** Primers used to confirm the presence of DNA of selected parasitic pathogens in ticks from six Caribbean territories.

Pathogen	Target gene	Primer design	Sequence (5′→3′)	Length (bp)	References
*Anaplasma*/ *Ehrlichia* spp.	16S rRNA	EHR1 ^∗^ EHR2 ^∗^ EHR3 ^∗∗^ EHR4 ^∗∗^	GAACGAACGCTGGCGGCAAGCAGTAYCGRACCAGATAGCCGCTGCATAGGAATCTACCTAGTAGAGTAYCGRACCAGATAGCCGC	693 592	[102]

*Babesia*/ *Theileria*/ *Hepatozoon* spp.	18S rRNA	BTH 18S 1St F ^∗^ BTH 18S 1St R ^∗^ BTH 18S 2Nd F ^∗∗^ BTH 18S 2Nd R ^∗^	GTGAAACTGCGAATGGCTCATTACAAGTGATAAGGTTCACAAAACTTCCCGGCTCATTACAACAGTTATAGTTTATTTGCGGTCCGAATAATTCACCGGAT	1500	[103]

*Babesia-Theileria* spp.	18S rRNA	BABGF2 BABGR2	GYYTTGTAATTGGAATGATGGCCAAAGACTTTGATTTCTCTC	559	[104]

*Hepatozoon canis*	18S rRNA	HepF ^∗^ HepR ^∗^ HepNF ^∗∗^ HepNR ^∗∗^	ATACATGAGCAAAATCTCAACCTTATTATTCCATGCTGCAGGGTATGGTATTGGCTTACCGCGAGCTTTTTAACTGCAACA	660 309	[105, 106]

*Rickettsia* spp.	*omp*B	Rc.rompB.4362p ^∗^ Rc.rompB.4836n ^∗^ Rc.rompB.4494p ^∗∗^ Rc.rompB.4762n ^∗∗^	GTCAGCGTTACTTCTTCGATGCCCGTACTCCATCTTAGCATCAGCCAATGGCAGGACTTAGCTACTAGGCTGGCTGATACACGGAGTAA	475 267	[107]

*Borrelia* spp.	flaB	FlaB280FFlaRL FlaB737F FlaLL	GCAGTTCARTCAGGTAACGGGCAATCATAGCCATTGCAGATTGTGCATCAACTGTRGTTGTAACATTAACAGG ACATATTCAGATGCAGACAGAGGT	645 407	[108]

*Mycoplasma* spp.	16S rRNA	Myco184‐F1 ^∗^ Myco1310‐R1 ^∗^ Myco322‐F2 Myco938‐R2	ACCAAGSCRATGATRGRTAGCTGGACRGGATTACTAGTGATTCCAACTTCAAGCCCATATTCCTACGGGAAGCAGCAGTCTCCACCACTTGTTCAGGTCCCCGTC	1127 500	[109]

^∗^Outer primer.

^∗∗^Inner primer; *Y*: T/C; *R*: A/G.

### 2.7. Phylogenetic Sequence Analysis

Sequence alignments were performed using the Muscle algorithm in MEGA 11 software [37, 38]. Maximum likelihood [39] and Neighbour‐joining [40] trees were generated by 1000 bootstrap repetitions [41] based on the Hasegawa–Kishino–Yano and Kimura‐2 parameter models in MEGA 11 [39, 42]. Trees were drawn to scale, with branch lengths in the same units as those of the evolutionary distances used to infer the phylogenetic trees. Sequences deposited into GenBank by this current study were marked with a black circle, square, triangle or diamond in the phylogenetic trees.

### 2.8. Statistical Analysis

Data collected were recorded in Microsoft Excel and coded for analysis in SPSS (IBM SPSS Statistics, Version 25). Data were displayed in tables and figures, and the chi‐square test of independence was used to determine the frequency of TBP infection of ixodid ticks in the territories sampled and any statistically significant differences in the detection frequencies of parasitic pathogens (independent variables) and the dependent variables (ticks and animal host: cattle and dogs) investigated. Statistical significance was set at *p* < 0.05.

## 3. Results

### 3.1. Prevalence and Diversity of TTBPs in Six Caribbean Territories

A total of 840 ticks (744 adults and 96 nymphs), composed of five tick species, were tested from a total of 111 cattle and 155 dogs in Barbados, Guyana, St. Kitts, St. Lucia, Tobago, and Trinidad. It should be noted that ticks were collected and tested only from cattle in St. Lucia; one *R. microplus* was found on a dog in Guyana, and at least one *Amblyomma* species was found in each territory sampled. Demographic data are shown in Table 2.

**Table 2:** Demographic data for the six territories sampled for ticks and their pathogens from cattle and dogs.

Cattle	Guyana	Trinidad	Tobago	Barbados	St. Lucia	St. Kitts	Total
Total number of farms	15	14	15	4	5	3	56

Total number of cattle	15	51	21	7	14	3	111

Total number of ticks	28	91	35	37	101	43	335

Tick species	23 *R.m*. 4 *A. caj*.	91 *R.m*.	36 *R.m*.	37 *R.m*.	97 *R.m*. 4 *A. var*.	41 *R.m*. 2 *A. var*.	325 *R.m*. 4 *A. caj*. 6 *A. var*.

**Dogs**	**Guyana**	**Trinidad**	**Tobago**	**Barbados**	**St. Lucia**	**St. Kitts**	**Total**

Total number of households	4	89	17	5	^a^	15	130

Total number of dogs	4	105	21	9	^a^	16	155

Total number of ticks	4	258	35	89	^a^	119	505

Tick species	3 *R.s*. 1 *R.m*.	238 *R.s*. 20 *A. ov*.	34 *R.s*. 1 *A. ov*.	87 *R.s*. 2 *A. v*ar.	^a^	119 *R.s*.	481 *R.s*. 21 *A. ov*. 2 *A. var*. 1 *R.m*.

Abbreviations: *A. caj*., *Amblyomma cajennense* s.l.; *A. ov*., *Amblyomma ovale*; *A. var*., *Amblyomma variegatum*; *R. m*., *Rhipicephalus microplus*; *R. s*., *Rhipicephalus sanguineus* s.l..

^a^Dogs not sampled.

Morphological identification of *R. microplus* (*n* = 326), *R. sanguineus* s.l. (*n* = 481), *A. variegatum* (*n* = 8) and *A. cajennense* s.l. (*n* = 4) were consistent with molecular identification using the BioMark system. Molecular identification of *A. ovale* (*n* = 21) was not conducted in this study due to resource and logistical constraints. However, morphological identification was deemed sufficient, as the specimens exhibited distinct and well‐characterised morphological features consistent with established taxonomic keys. Pathogen diversity for each territory by tick species is shown in Figure 1a,b.

Figure 1(a) Total number of pathogen species (*N* = 18), detected in ticks infesting dogs from five Caribbean territories. (b) Total number of pathogen species (*N* = 13), detected in ticks infesting cattle from six Caribbean territories.

**Figure:**
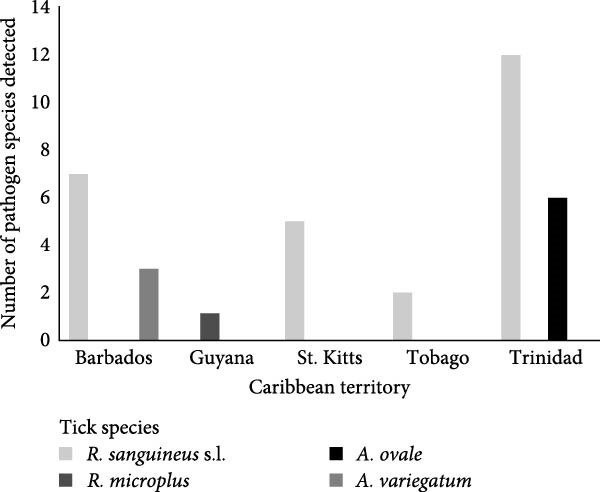
(a)

**Figure:**
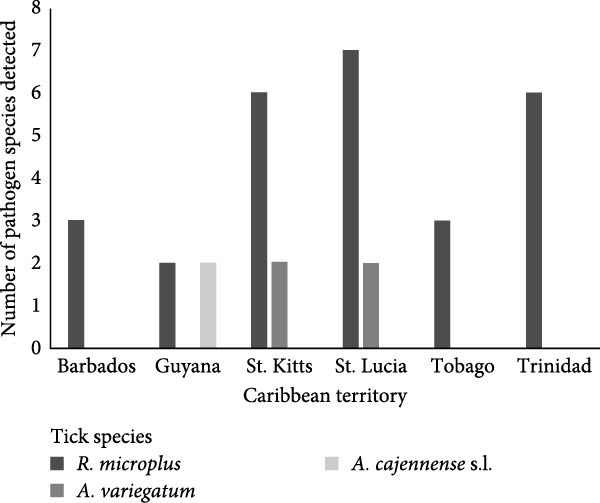
(b)

#### 3.1.1. TBPs in Ticks Removed From Dogs

Ticks tested from dogs (*N* = 505) across five of the six Caribbean territories revealed a high diversity of pathogens (Table 3). It should be noted that in St. Lucia, focus was placed on sampling ticks from cattle only. The most predominant tick species found on dogs was *R. sanguineus* s.l. (*n* = 481), followed by *A. ovale* (*n* = 21) and *A. variegatum* (*n* = 2). One *R. microplus* tick was found on a dog from Guyana.

**Table 3:** Frequency distribution of pathogens found in ticks removed from dogs with single infections^a^.

Pathogens	*Rhipicephalus sanguineus* s.l.^b^ (%) (*N* = 481)				*Amblyomma* *ovale* (%) (*N* = 21)	Number (%) *N* = 505
	Barbados (*n* = 87)	St. Kitts (*n* = 119)	Tobago (*n* = 34)	Trinidad (*n* = 238)	Trinidad (*n* = 20)	
*Anaplasma marginale*	0	0	0	1 (0.4)	0	1 (0.2)
*Anaplasma platys*	1 (1.1)	5 (4.2)	0	8 (3.4)	0	14 (2.8)
*Borrelia* spp.	0	0	0	1 (0.4)	0	1 (0.2)
*Ehrlichia canis*	1 (1.1)	0	0	0	0	1 (0.2)
*Ehrlichia* spp.	8 (9.2)	0	1 (2.9)	0	0	9 (1.8)
*Ehrlichia ruminantium*	8 (9.2)	0	0	0	0	8 (1.6)
*Mycoplasma wenyonii*	1 (1.1)	0	0	1 (0.4)	0	2 (0.4)
*Rickettsia* spp.	0	4 (3.4)	0	1 (0.4)	0	5 (1.0)
*Rickettsia africae*	2 (2.3)	0	0	0	0	2 (0.4)
*Rickettsia felis*	1 (1.1)	0	0	8 (3.4)	1 (5)	10 (2.0)
*Babesia bigemina*	0	0	0	0	1 (5)	1 (0.2)
*Babesia bovis*	0	0	0	1 (0.4)	0	1 (0.2)
*Babesia vogeli* ^c^	0	0	0	1 (0.4)	0	1 (0.2)
*Hepatozoon canis*	0	0	6 (17.6)	9 (3.8)	0	15 (3.0)
*Theileria mutans*	0	7 (5.9)	0	0	0	7 (1.4)
*Theileria velifera*	0	2 (1.7)	0	1 (0.4)	0	3 (0.6)
*Theileria equi*	0	0	0	1 (0.4)	0	1 (0.2)
Total number of single infected ticks	22 (25.3)	18 (15.1)	7 (20.6)	33 (13.9)	2 (10.0)	83^c^ (16.4)

^a^Territories with no detection of tick pathogens have been omitted, for example, Guyana, *R. sanguineus* s.l. (*n* = 3), Tobago, *A. ovale* (*n* = 1).

^b^Dogs not sampled in St. Lucia.

^c^One *R. microplus* infected with *B. vogeli* removed from a dog from Guyana, included in the table total (*n* = 83), only.

Overall, in the five territories, single infections were detected in 16.4% (83/505) of ticks, with *H. canis* (*n* = 15), *Anaplasma platys* (*n* = 14), *Ehrlichia* spp. (*n* = 9) and *R. felis* (*n* = 10), being the most frequently detected. The distribution of these pathogens by territory and tick species is shown in Table 3. Barbados contributed the highest frequency of singly infected *R. sanguineus* s.l. ticks 25.3% (22/87); however, the highest diversity of pathogens in *R. sanguineus* s.l. with single infections was detected in Trinidad (*n* = 11) with an infection rate of 13.9% (33/238), compared to seven TBP species in Barbados. Of note was the detection of *E. ruminantium* in 9.2% (8/87) and *R. africae* in 2.3% (2/87) of *R. sanguineus* s.l. ticks from Barbados. *A. ovale* was found on dogs from Trinidad (*n* = 20) and Tobago (*n* = 1) only. Two pathogens were detected in singly infected *A. ovale* ticks from Trinidad, but none from Tobago. Four ticks (*R. sanguineus* s.l.; *n* = 3 and *R. microplus*; *n* = 1) were tested from dogs in Guyana. Only one pathogen, *B. vogeli*, was detected in the *R. microplus* tick.

Overall, dual infections were identified in 1.4% (7/505) of ticks removed from dogs in three of the territories sampled (Table S2). The rate of dual infections ranged from 1.1% to 1.7% in *R. sanguineus* s.l. ticks from Barbados, Trinidad and St. Kitts, respectively, while 50% (1/2) of *A. variegatum* from Barbados were infected with two pathogens. Triple infections were found in only three ticks from Trinidad (*n* = 2) and Barbados (*n* = 1) (Table S3). The chi‐square test for independence showed that the frequency of single vs co‐infections for *R. sanguineus* s.l. (*p* = 0.94, χ^2^ 8 *df*), was independent of the territory sampled.

#### 3.1.2. DNA Sequencing and Phylogenetic Diversity of TBPs in Ticks From Dogs

Pathogens detected in this study were confirmed by conventional PCR followed by amplicon sequencing. Different gene targets from those used in the BioMark system were employed to demonstrate species specificity and sensitivity of all primers and probe sets (Table 1). The 16S rRNA gene for *Anaplasma* and *Ehrlichia* spp. and the 18S rRNA gene for *Babesia*, *Theileria* and *Hepatozoon* spp. were selected for confirmation of these species based on literature reviews when the tools were developed [30, 32]. BLAST analysis confirmed the presence of *A. marginale* from Trinidad (accession number PP933767) with 100% homology to a sequence from the USA (accession number AF311303) (Table 4). Sequence analysis also confirmed the presence of *E. canis*, *H. canis*, *R. felis*, *B. bigemina*, *B. bovis*, *B. vogeli*, and *T. equi* with sequences previously published in GenBank, ranging from 99.5% to 100% homology. Sequence analysis for some TBPs could not be performed due to the poor amplification of nucleic acids.

**Table 4:** Homology between deposited sequences and reference sequences in GenBank.

Vector‐borne pathogens	Length	Identity (%)	Query cover (%)	Reference sequence	Country	Deposited sequence	Country
*Anaplasma marginale*	634 426 342	100 99.8 100	100 100 100	AF311303 OQ362281 KC189844	USA Cuba South Africa	PP933767 PP933765 PP933762	Trinidad Tobago Tobago

*Ehrlichia canis*	544 193	100 100	100 100	OR291155 OP164605	Cuba Thailand	PP933757 PP933758	Trinidad Tobago

*Ehrlichia* spp.	405	100	100	OP047994	China	PP933760	Trinidad

*Rickettsia felis*	266	100	100	GU182892	Sweden	PQ096847	Trinidad

*Babesia bigemina*	212	99.5	100	KU206296	Uganda	PP824977	Trinidad

*Babesia bovis*	191	100	100	MH046910	USA	PP824975	Trinidad

*Babesia vogeli*	162 888	100 100	100 100	AY371197 MK881091	Egypt China	PP824971 PP824979	Trinidad Trinidad

*Hepatozoon canis*	279 344 86 207	100 99.8 100 100	100 100 100 100	AF176835 LC775880 MK827809 AF176835	USA Iraq Poland USA	PP824973 PP824972 PP824978 PP824980	Trinidad Trinidad Trinidad Tobago

*Theileria equi*	395	100	100	MH100725	Paraguay	PP824976	Trinidad

**Figure 2:**
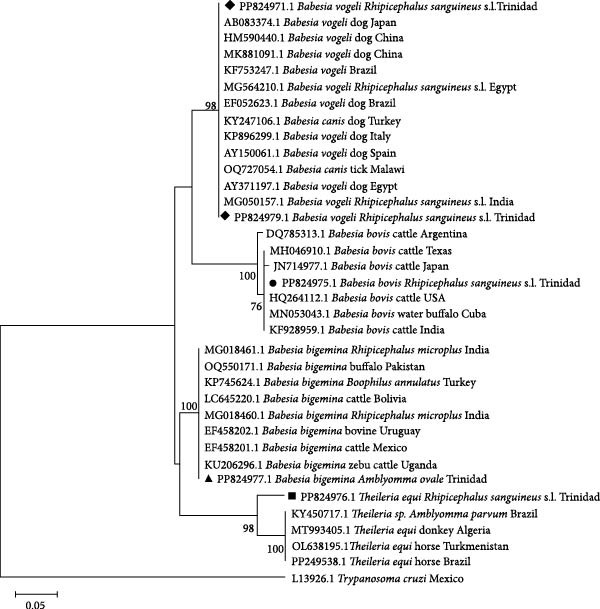
Phylogenetic tree of selected representatives of *Babesia* and *Theileria* spp. inferred from 18S rRNA. Sequences from this study are indicated by a black diamond, circle, triangle or square. The evolutionary history was inferred using the maximum likelihood method and Kimura‐2 parameter model [39]. The tree with the highest log likelihood (−655.86) is shown. The percentage of trees in which the associated taxa clustered together is shown below the branches. Initial trees for the heuristic search were obtained by applying the neighbour‐Joining method to a matrix of pairwise distances estimated using the maximum composite likelihood (MCL) approach. The tree is drawn to scale, with branch lengths measured in the number of substitutions per site. This analysis involved 36 nucleotide sequences. All positions with less than 95% site coverage were eliminated; that is, fewer than 5% alignment gaps, missing data, and ambiguous bases were allowed at any position (partial deletion option). There was a total of 159 positions in the final dataset. Evolutionary analyses were conducted in MEGA11 [38].

The 18S rRNA gene was used to perform phylogenetic and genetic distance analyses of *Babesia* and *Theileria* species found in this study, compared to other sequences from various members of the genera in different territories. *Babesia bovis* (accession number PP824975, black circle), *B. bigemina* (accession number PP824977, black triangle) and *B. vogeli* (accession numbers PP824971 and PP824971, black diamonds) clustered with their respective species with no major sub‐clustering (Figure 2). Additionally, the *T. equi* sequence (accession number PP824976, black square) clustered basally to the *T. equi* clade.

**Figure 3:**
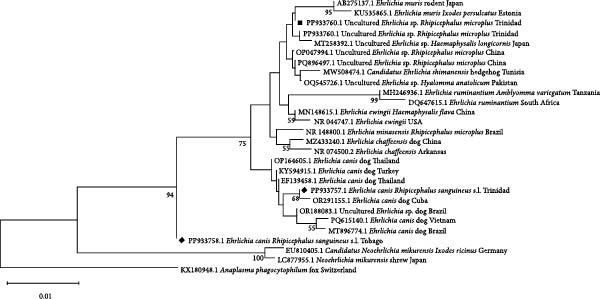
Phylogenetic tree of selected representatives of *Ehrlichia* spp. inferred from 16S rRNA. Sequences from this study are indicated by a black diamond or square. The evolutionary history was inferred using the neighbour‐joining method [40]. The optimal tree is shown. The percentage of replicate trees in which the associated taxa clustered together in the bootstrap test (1000 replicates) are shown below the branches [41]. The tree is drawn to scale, with branch lengths in the same units as those of the evolutionary distances used to infer the phylogenetic tree. The evolutionary distances were computed using the Kimura 2‐parameter method [39] and are in the units of the number of base substitutions per site. This analysis involved 28 nucleotide sequences. All ambiguous positions were removed for each sequence pair (pairwise deletion option). There were a total of 1264 positions in the final dataset. Evolutionary analyses were conducted in MEGA11 [38].

**Figure 4:**
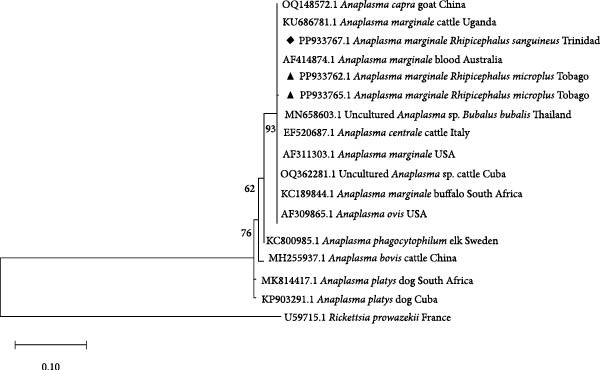
Phylogenetic tree of selected representatives of *Anaplasma* spp. inferred from 16S rRNA. Sequences from this study are indicated by a black diamond or triangle. The evolutionary history was inferred by using the maximum likelihood method and the Hasegawa–Kishino–Yano model [42]. The tree with the highest log likelihood (−878.40) is shown. The percentage of trees in which the associated taxa clustered together is shown below the branches. Initial trees for the heuristic search were obtained automatically by applying the maximum parsimony method. The tree is drawn to scale, with branch lengths measured in the number of substitutions per site. This analysis involved 18 nucleotide sequences. All positions containing gaps and missing data were eliminated (complete deletion option). There were a total of 323 positions in the final dataset. Evolutionary analyses were conducted in MEGA11 [38].

To investigate the genetic relationships of the Anaplasmataceae (*Anaplasma* and *Ehrlichia* spp.), a portion of the 16S rRNA gene was used. The *A. marginale* sequence from Trinidad (accession number PP933767, black diamond) clustered in the same clade as other *A. marginale* sequences from the US, Africa, Asia and Australia (Figure 3). *E. canis* (accession number PP933757, black diamond) clustered with *E. canis* sequences from territories including Cuba, while the sequence with accession number PP933758 (black diamond) clustered basally to the clades containing *E. canis* and an uncultured *Ehrlichia* spp. The uncultured *Ehrlichia* spp. (black square), clustered with the clade containing other uncultured *Ehrlichia* spp., but sister to *E. canis* (Figure 4).

#### 3.1.3. TBPs in Ticks Removed From Cattle

A total of 335 ticks were tested from cattle from the six territories sampled in this study. *R. microplus* (*n* = 325), was the predominant species in all territories, with fewer numbers of *A. variegatum* (*n* = 6) and *A. cajennense* s.l. (*n* = 4) (Table 5). Overall, single infections were detected in 21.8% (73/335) of ticks, with *A. marginale*, 8.4% (28/335) being the most frequently detected, particularly in Trinidad, 15.4% (14/91) and St. Kitts, 12.1% (5/41). Other frequently detected pathogens included *Ehrlichia* spp. (*n* = 17), *Rickettsia* spp. (*n* = 7) and *R. africae* (*n* = 6). The distribution of pathogens by territory and tick species is displayed in Table 5. The highest diversity of TBPs was detected in *R. microplus* from both St. Lucia (*n* = 5) and Trinidad (*n* = 5), with the lowest diversity from Guyana (Figure 1b). Only one TBP, *A. marginale*, was amplified from a *R. microplus* tick from Guyana.

**Table 5:** Frequency distribution of pathogens found in ticks removed from cattle with single infections.

Pathogens	*Rhipicephalus microplus* (%) (*N* = 326)						*Amblyomma variegatum* (%) (*N* = 8)		*Amblyomma* *cajennense* (%) (*N* = 4)	Number (%) *N* = 335
	Barbados (*n* = 37)	Guyana (*n* = 23)	St. Kitts (*n* = 41)	St. Lucia (*n* = 97)	Tobago (*n* = 36)	Trinidad (*n* = 91)	St. Kitts (*n* = 2)	St. Lucia (*n* = 4)	Guyana (*n* = 4)	
*Anaplasma marginale*	3 (8.1)	1 (4.3)	5 (12.1)	2 (2.1)	3 (8.3)	14 (15.4)	0	0	0	28 (8.4)
*Ehrlichia* spp.	1 (2.7)	0	1 (2.4)	5 (5.2)	0	10 (11)	0	0	0	17 (5.1)
*Mycoplasma wenyonii*	0	0	0	3 (3.1)	0	0	0	0	0	3 (0.9)
*Rickettsia* spp.	0	0	4 (9.8)	1 (1.0)	0	0	0	2 (50)	0	7 (2.1)
*Rickettsia africae*	0	0	4 (9.8)	1 (1.0)	0	0	0	1 (25)	0	6 (1.8)
*Rickettsia felis*	0	0	0	0	3 (8.3)	0	0	0	0	3 (0.9)
*Rickettsia parkeri*	0	0	0	0	0	0	1 (50)	0	0	1 (0.3)
*Babesia bigemina*	0	0	0	0	0	1 (1.1)	0	0	0	1 (0.3)
*Babesia bovis*	0	0	0	0	0	1 (1.1)	0	0	0	1 (0.3)
*Babesia vogeli*	0	0	0	0	0	1 (1.1)	0	0	1 (25)	2 (0.6)
*Hepatozoon canis*	0	0	0	0	1 (2.8)	0	0	0	0	1 (0.3)
*Theileria mutans*	0	0	2 (4.9)	0	0	0	0	0	0	2 (0.6)
*Theileria velifera*	1 (2.7)	0	0	0	0	0	0	0	0	1 (0.3)
Total number of single infected ticks	5 (13.5)	1 (4.3)	16 (39.0)	12 (12.4)	7 (19.4)	27 (29.7)	1 (50.0)	3 (75.0)	1 (25.0)	73 (21.8)

Dual infections were detected in 6.3% (21/335) of ticks removed from cattle in five of the six territories sampled (Table S4). The highest frequency was detected in *R. microplus* (*n* = 19), with infection rates ranging from 3.1% to 10.8% based on territory. Notably, *E. ruminantium* and *R. africae* were found in *A. variegatum* from Barbados, 50% (1/2), whilst dual infection of 25% (1/4) in *A. cajennense* s.l. tick with *A. marginale* and *B. vogeli* was detected in Guyana. Triple infections were detected in two *R. microplus* ticks from St. Kitts (*n* = 1) and St. Lucia (*n* = 1). (Table S5). The chi‐square test for independence showed that the frequency of single vs co‐infections for *R. microplus* ticks was significantly associated with territories sampled (*p* = 0.002, χ^2^ 8 df).

#### 3.1.4. DNA Sequencing and Phylogenetic Diversity of TBPs in Ticks From Cattle

Sequences obtained for pathogens detected in ticks removed from cattle in this study were also deposited in GenBank. The highest matches for the sequences submitted from this study are shown in Table 4. Sequence analysis confirmed the presence of *A. marginale* from Tobago (accession numbers PP933762 and PP933765) with 100% homology to a sequence from Cuba (OQ362281) and South Africa (KC189844), respectively. Both Tobago sequences (black triangles) clustered with other *A. marginale* species globally (Figure 3). The uncultured *Ehrlichia* spp. (PP933760, black square), from a *R. microplus* tick from Trinidad, clustered with the clade containing other uncultured *Ehrlichia* spp. globally (Figure 4).

## 4. Discussion

Ticks play a major role in the epidemiology of animal and human diseases globally, including in the Caribbean region. To the best of our knowledge, this study represents the first large‐scale survey utilising a high‐throughput microfluidic real‐time PCR system for detecting bacterial and protozoan TBPs of ticks in several territories in the English‐speaking Caribbean. This system has been recently used to detect TBPs in ticks and dogs in Cuba and the French Caribbean Islands (Guadeloupe and Martinique) [30, 33].

*A. marginale*, the causative agent of bovine anaplasmosis, is endemic in the Americas, including the Caribbean region, Africa, Asia, Australia and Europe [43, 44]. This TBP has been detected in cattle ticks from all six territories sampled in this study, which is consistent with its detection from cattle in the region [6, 30, 45]. Published reports of *A. marginale* in the blood of cattle and ticks are, however, lacking for Guyana. Apart from cattle ticks, *A. marginale* was also detected in three *R. sanguineus* s.l. and one *A. ovale* tick removed from dogs in Trinidad. Whilst the cattle tick is a common vector of *A. marginale*, biological transmission can also be effected by at least 20 species of ticks, including *R. sanguineus* s.l., which supports our findings [46, 47]. *A. marginale* was detected in 2.1%–15.4% of the singly infected *R. microplus* ticks from the six territories in our study. A previous study in the French West Indies also reported less than 5% detection of *A. marginale* in *R. microplus* from Guadeloupe, but ~ 40% infected cattle ticks from Martinique [30].

*A. platys* (1.1%–4.2%) was detected in *R. sanguineus* s.l. from three of the six territories tested. The highest frequency was reported in dog ticks from St. Kitts, followed by Trinidad, then Barbados. This rickettsial pathogen is endemic to the canine population in the Caribbean and is also known to affect cats, cattle, and humans [48–52]. Previous studies have shown that the prevalence of *A. platys* in dogs from the Caribbean ranged from ~ 5% to 25%. [21, 23, 33, 53, 54]. However, the frequency of this TBP from ticks was lower when compared to their canine hosts. A recent study in Cuba detected *A. platys* in 9.3% of the sampled brown dog ticks, which is comparable to our findings. Concrete evidence, however, is still needed to ascertain *R. sanguineus* s.l. as a biological vector for *A. platys*.

Canine monocytic ehrlichiosis, caused by *E. canis* and transmitted by *R. sanguineus* s.l., has been reported in ticks and companion animals in the Caribbean [14, 21, 53–55]. This TBP was found in *R. sanguineus* s.l. (*n* = 3) and *A. ovale* (*n* = 1), removed from dogs in our study, some of which were co‐infested by both ticks. Previous findings of this TBP in *A. ovale* were also reported in Mexico [56]. This, however, does not implicate *A. ovale* as a competent vector of *E. canis;* thus, infection studies are needed to justify this tick as a potential vector. Sequencing and phylogenetic analysis indicated the clustering of *E. canis* and the uncultured *Ehrlichia* spp. from our study in separate sister clades, indicating that both species may be closely related. Future work involving the sequencing of other genes is necessary to support our findings.

An important finding of this study was the detection of *E. ruminantium* DNA, by microfluidic analysis only, from ticks harvested from two dogs. One dog was co‐infested with *A. variegatum* and *R. sanguineus* s.l. while the other was infested with *R. sanguineus* s.l. only. This is the first report of the detection of *E. ruminantium* DNA in Barbados. *A. variegatum* was reported in Barbados in 1984 (two male ticks in the parish of St. Lucy) and 1990–1991 in three additional parishes, but the country was certified ’provisionally free’ in 2003, with periodic reports of hot spots [57]. *Amvblyomma variegatum* primarily feeds on ruminants, including cattle; however, other mammals, including dogs, may serve as hosts [58]. It is also important to note that *A. variegatum* was not detected on cattle from Barbados in our study, nor were there any reports of clinical signs of bovine ehrlichiosis on the island. We can postulate that the presence of *E. ruminantium* DNA in both tick species in our study may have occurred during co‐feeding with the canine host, acting as a bridge between the two tick species [59]. Further investigations involving *E. ruminantium* validation via sequencing and more in‐depth surveillance of *A. variegatum* and its pathogens in Barbados are required to mitigate any possible future issues in the ruminant industry.

A *Borrelia* spp. has been identified for the first time in St. Lucia (*R. microplus*; 1%), St. Kitts (*R. sanguineus* s.l.; 0.8%) and Trinidad (*R. sanguineus* s.l.; 0.4%) using the BioMark system. Unfortunately, further sequencing was not done due to the unavailability of the tick DNA. In two cases, ticks were co‐infected with at least one other TBP. *Borrelia* spp. have been reported in ticks from the French Caribbean islands, Guadeloupe and Martinique, with sequences closely related to *Borrelia anserina* and a relapsing fever *Borrelia* spp [30, 60–62]. The pathogenicity of our *Borrelia* spp. is unknown. It should, however, be noted that cases of erythema‐like skin lesions have been reported in human patients in the Caribbean, including Trinidad, but the organisms were not isolated in any of these cases [62].

*R. africae*, the causative agent of African tick bite fever (ATBF), which is potentially fatal to humans, was detected in *A. variegatum* ticks from Barbados, St. Lucia and St. Kitts. *A. variegatum* is the vector of *R. africae* and has persisted on St. Kitts and St. Lucia for at least five decades, even after eradication efforts [57, 63]. Further, *R. africae* has been previously reported in the Caribbean, including some of the territories sampled in our study [28–30, 64, 65]. One such study detected *R. africae* in 7%–50% of bont ticks, *A. variegatum* [29], while a more recent study detected this pathogen in almost 96% of the tick DNA tested [30]. The 25%–50% infection rate of *A. variegatum* with *R. africae* in our study may be due to the small sample size. It should, however, be noted that this tick is both a competent vector and reservoir of *R. africae* with up to 100% transmission (trans‐ovarial and trans‐stadial) rates [29, 66]. *R. africae* was also detected in *R. microplus* at lower frequencies from St. Kitts and St. Lucia, and one brown dog tick from Barbados. Although these ticks are not known to be competent vectors of *R. africae*, this pathogen has been previously detected in cattle ticks [67]. Since *R. microplus* and *A. variegatum* may feed on the same animal at once, as was observed in our study and a previous study [30], these ticks may have acquired *R. africae* through co‐feeding with *A. variegatum* or the infected blood meal from its vertebrate host [68]. A similar situation may have existed to explain the presence of *R. africae* in the brown dog tick.

An important finding was the detection of *R. parkeri* in two *A. variegatum* ticks from St. Kitts and one co‐infected TBT from Barbados. To the best of our knowledge, this is the first published report of *R. parkeri* in the geographic region studied. However, further sampling and molecular analyses should be undertaken to confirm the presence of this pathogen in this region. *Rickettsia parkeri* has been implicated in human cases of spotted fever in Brazil, Uruguay, and Argentina and is the second most prevalent TBP in the spotted fever group rickettsiae (SFGR) in the Americas [69, 70]. Considering that human patients typically suffer from fevers, rash, and myalgias, it may sometimes be misdiagnosed as dengue, leptospirosis, or other infectious diseases with similar symptoms [71, 72]. Therefore, surveillance and detection of pathogens in the Caribbean tick population can function as a sentinel system providing early warning signals of potential risk to public health.

The cat flea, *Ctenocephalides felis*, is currently the only known biological vector of *R. felis*; however, there is molecular evidence of this pathogen being present in other flea species, ticks and mites [73]. The detection of *R. felis* in ticks in this current study highlights the potential role of *R. sanguineus* s.l. and *R. microplus* as vectors of an SFGR in Barbados, Trinidad and Tobago [74, 75]. The findings of *R. felis* in ticks are consistent with previous studies in Cuba that detected this pathogen in *R. sanguineus* s.l. from stray and owned dogs and also *Dermacentor nitens* from horses [76]. It should, however, be noted that the *R. felis* strain detected in this study shared 100% identity with strains detected in India, Cuba, and Sweden. The latter was associated with two human cases of subacute meningitis [77]. Therefore, further research is needed to clarify the role of *R. sanguineus* s.l. and *R. microplus* as competent vectors of *R. felis* in the Caribbean.

The DNA of an unspecified *Rickettsia* spp. was also amplified in ticks removed from dogs and cattle in St. Kitts, St. Lucia, and Trinidad. Pathogenic rickettsias were also detected in these same territories. We propose that this microorganism may be a tick endosymbiont, but further work is needed to elucidate this hypothesis [78].

The haemotrophic mycoplasma, *Mycoplasma wenyonii*, formerly known as *Eperythrozoon wenyonii* [79] was detected in *R. sanguineus* s.l. and *R. microplus* (0.4%–3.1%), from three of the six territories by real‐time PCR. This emerging bacterial pathogen affects animals, especially cattle and humans, and is believed to be transmitted by haematophagous arthropods, including ticks [79–81]. This pathogen produces a bacteraemia and mild anaemia but may cause more severe disorders like haemolytic anaemia in stressed animals [82]. To date, *M. wenyonii* has only been reported in cattle and water buffalo from Cuba [83], while *Mycoplasma haemocanis*, *Candidatus Mycoplasma haematoparvum*, *Mycoplasma haemofelis*, and *Candidatus Mycoplasma haemominutum* have been reported in dogs and cats in other Caribbean islands [84, 85]. Since the epidemiology of bovine haemotropic mycoplasmas is still poorly understood, future studies on the vector and host populations should be undertaken to determine their impact on the livestock industry in the region.

*Babesia bovis* and *B. bigemina*, transmitted by *R. microplus*, are endemic in the Caribbean and cause significant global losses to the livestock industry [6, 86–88]. Previous seroprevalence studies in the region have reported infection rates as high as 69% in cattle. A study in Guadeloupe and Martinique detected *B. bigemina* in ~ 1% and 12% of *R. microplus* ticks, respectively [30]. The low detection rate in our study (1.1% in *R. microplus* ticks) was similar to the findings in Guadeloupe [30]. This TBP was also found in a single *A. ovale* tick removed from a dog in Trinidad. Interestingly, *Babesia bovis* was only amplified in two *R. sanguineus* s.l. ticks removed from two separate dogs from Trinidad. A possible explanation for this is that these ticks may have fed on a ruminant infected with *B. bovis* or co‐fed with infected *R. microplus* ticks. This is plausible since the canine hosts were sampled from locations with tick‐infested cattle. The low infection rates of *B. bigemina* and *B. bovis* in our current study are not known; however, previous studies proposed that further investigations are needed to better understand the efficiency of acquisition and transmission of these TBPs from *R. microplus* to their vertebrate hosts [89, 90].

*B. vogeli* is transmitted by *R. sanguineus* s.l. and is associated with disease in dogs. However, only one *R. sanguineus* s.l., two *R. microplus*, and one *A. cajennense* s.l. were infected with this TBP. Previous studies have also detected this parasite in the blood of cats, donkeys, goats, and sheep in the Caribbean [84, 88]. A recent study in Tobago found *B. vogeli* in *R. sanguineus* s.l. and *A. ovale*, both of which were removed from dogs. In our case, three of the four infected ticks were removed from cattle. A possible explanation for the presence of *B. vogeli* in *R. microplus* and *A. cajennense* s.l. could be due to these ticks acquiring a blood meal from an infected dog. Although this TBP was amplified from three different tick species, sequence and phylogenetic analyses revealed a high level of conservation in *B. vogeli*.

This is the first report of *T. mutans* and *T. velifera* in cattle and dog ticks in the English‐speaking Caribbean. Serological evidence of these pathogens has been reported in Cuba, Guadeloupe, and Martinique [55, 91–93]. Both TBPs cause benign theileriosis, which may explain the lack of data available for these pathogens throughout the Caribbean.

*H. canis* was this study’s most frequently detected TBP of protozoan origin in brown dog ticks. Similar findings were observed in a recent study from Cuba, where *H. canis* was the most common pathogen detected in *R. sanguineus* s.l. ticks [33]. Interestingly, this parasite was only amplified in ticks from Trinidad and Tobago, although the vector, *R. sanguineus* s.l. was found in all territories sampled, and there have been previous reports of *H. canis* in other Caribbean islands [14, 21, 23, 53, 94]. Notably, *A. ovale* was found only on dogs in Trinidad and Tobago and is also implicated in the transmission of *H. canis* [95]. The relatively high prevalence of *H. canis* in this study raises concerns, as it causes clinical disease in dogs, posing a risk to the canine population on both islands.

*Theileria equi* was detected in one *R. sanguineus* s.l. tick from Trinidad. This TBP causes equine piroplasmosis and is listed as a notifiable disease by the World Organisation for Animal Health (WOAH). There is serological and molecular evidence of *T. equi* in the Caribbean (Dominica, St. Kitts & Nevis, and Trinidad) (95–97); however, this is the first report of *T. equi* DNA recovered from ticks in Trinidad. Although many species of ixodid ticks have been identified (naturally or experimentally) as vectors of *T. equi*, *Dermacentor nitens* is thought to be the primary vector in the Caribbean [96, 97].

The nature of ticks feeding on the blood of multiple hosts during their lifespan could predispose them to co‐infections with several pathogens, thereby increasing the chances of co‐transmission to humans and animals [98]. This may alter clinical manifestations, disease severity and clinical outcomes in the affected host, at times with fatal consequences [99]. Co‐infections have been previously reported in ixodid ticks, including *A. variegatum*, *R. sanguineus* s.l. and *R. microplus*. [30, 33, 98, 100, 101]. In our study, co‐infections with at least two pathogens were detected in ~ 4% of infected ticks. The highest frequencies of co‐infection were in *R. microplus* and *R. sanguineus* s.l. ticks. However, much higher co‐infection rates were observed in *R. microplus* ticks removed from cattle in Guadeloupe and Martinique and brown dog ticks from owned dogs in Cuba [30, 33]. These observations highlight the efficiency of these ticks as possible reservoirs and potential vectors of several TBPs in the Caribbean.

## 5. Conclusions

This study emphasises the importance of large‐scale molecular investigations of ticks and the pathogens they harbour, using high‐throughput real‐time microfluidic PCR systems, especially in tropical regions with limited surveillance, reporting and diagnostic capabilities. Our findings highlight the high diversity of TBPs in ticks infesting cattle and dogs, thereby broadening the findings of the previous study undertaken in the French Antilles. This study also emphasises the importance of raising awareness from a One‐Health perspective among veterinarians, farmers, pet owners and medical doctors of the clinical signs and risks associated with TBDs in the Caribbean. Sequencing and phylogenetic analysis provided further insight into the identity of these TBPs and their relatedness globally. The co‐infections observed in ticks predominantly found on livestock and pet dogs highlight the need for improved point‐of‐care diagnostics using multiplex diagnostic platforms. Simultaneous detection of pathogens using this platform can serve as an early warning system for the detection of new and re‐emerging pathogens of economic and public health importance. We recommend that authorities in islands such as Barbados should pay close attention to the detection of heartwater disease and conduct syndromic surveillance activities on livestock in addition to active surveillance for the TBT. Development of multiplex diagnostics and regular vector surveillance should be prioritised in the Caribbean region as we strive to develop and sustain food security, particularly in the livestock industry.

## Ethics Statement

This study was approved by the Ethics Committee of the Faculty of Medical Sciences, the University of the West Indies (Approval Number CEC206/05/16). Consent to collect blood samples and ticks was obtained from the Chief Veterinary Officers and owners of the dogs and cattle in the territories sampled.

## Conflicts of Interest

The authors declare no conflicts of interest.

## Funding

This research was funded by the University of the West Indies, Campus Research and Publication Fund (Grant CRP.5.Jun17.28), the MALIN project ’Surveillance, diagnosis, control and impact of infectious diseases of humans, animals and plants in tropical islands’ supported by the European Union in the framework of the European Regional Development Fund (ERDF), the Regional Council of Guadeloupe (Grant 2015‐FED‐186) and ANSES and CIRAD PHD Grant (Grant AO‐20152017).

## Supporting Information

Additional supporting information can be found online in the Supporting Information section.

## Supporting information


**Supporting Information** Table S1: List of pathogens, tick species, targets, primers/probes, and positive controls and references used in this study. Table S2: Frequency distribution of pathogens found in dual‐infected ticks removed from dogs. Table S3: Frequency distribution of pathogens found in ticks removed from dogs with triple infections. Table S4: Frequency distribution of pathogens found in dual‐infected ticks removed from cattle. Table S5: Frequency distribution of pathogens found in ticks removed from cattle with triple infections.

## Data Availability

The original contributions presented in the study are included in the article/supporting information/online repositories. Further inquiries can be directed to the corresponding authors.
