# Supplementary material for: Diversity of Ticks in the Caribbean Region and Detection of Their Pathogens Using BioMark Technology
Source: Transbound Emerg Dis. 2025 Dec 5;2025:8135946. doi: 10.1155/tbed/8135946 (PMC12707301; doi:10.1155/tbed/8135946)
Supplement: Supplementary file 1 — Supporting Information Table S1: List of pathogens, tick species, targets, primers/probes, and positive controls and references used in this study. Table S2: Frequency distribution of pathogens found in dual‐infected ticks removed from dogs. Table S3: Frequency distribution of pathogens found in ticks removed from dogs with triple infections. Table S4: Frequency distribution of pathogens found in dual‐infected ticks removed from cattle. Table S5: Frequency distribution of pathogens found in ticks removed from cattle with triple infections. [file TBED-2025-8135946-s001.docx]

Supplementary Material

**Supplementary Table 1.** List of pathogens, tick species, targets, primers/probes, and positive controls and references used in this study.

| **Microorganisms** | | | **Gene**  **Target** | | **Design Name** | | | | | | **Sequence (5’ 🡪 3’)** | **Amplicon size (bp)** | **Positive controls** | | **Reference** |
| --- | --- | --- | --- | --- | --- | --- | --- | --- | --- | --- | --- | --- | --- | --- | --- |
| *Rickettsia*spp. | | | gltA | | Rick_spp_gltA_F  Rick_spp_gltA_R  Rick_spp_gltA_P | | | | | GTCGCAAATGTTCACGGTACTT  TCTTCGTGCATTTCTTTCCATTG TGCAATAGCAAGAACCGTAGGCTGGATG | | 78 | **, Culture of *R. slovaca* | | (30) |
| *Rickettsia akari* | | | ompB | | Ri_aka_ompB_F  Ri_aka_ompB_R  Ri_aka_ompB_P | | | | | GTGCTGTTGCAGGTGGTAC  TAAAGTAATACCGTGTAATGCAGC ATTACCAGCACCGTTACCTATATCACCGG | | 101 | Culture | | (30) |
| *Rickettsia andeanae* | | | ompB | | Ri_and_ompB_F  Ri_and_ompB_R  Ri_and_ompB_P | | | | | GGCGGACAGGTAACTTTTGG  AAGGATCATAGTATCAGGAACTG  ACACATAGTTGACGTTGGTACAGACGGTAC | | 165 | Naturally infected tick (unidentified spp.) | | (30) |
|  | | |  | | Ri_ma_ITS_F | | | | | GTTATTGCATCACTAATGTTATACTG | |  |  | | (32) |
| *Rickettsia massiliae* | | | ITS | | Ri_ma_ITS_R | | | | | GTTAATGTTGTTGCACGACTCAA | | 128 | Culture | |  |
|  | | |  | | Ri_ma_ITS_P | | | | | TAGCCCCGCCACGATATCTAGCAAAAA | |  |  | |  |
|  | | |  | | Ri_ri_ITS_F | | | | | TCTACTCACAAAGTTATCAGGTTAA | |  |  | | (32) |
| *Rickettsia rickettsii* | | | ITS | | Ri_ri_ITS_R | | | | | CCTACGATACTCAGCAAAATAATTT | | 124 | Plasmid | |  |
|  | | |  | | Ri_ri_ITS_P | | | | | TCGCTGGATATCGTTGCAGGACTACAG | |  |  | |  |
|  | | |  | | Ri_co_sca1_F | | | | | GTAGATGCTTCATAGAATACTGC | |  | Naturally infected | | (30) |
| *Rickettsia conorii* | | | sca1 | | Ri_co_sca1_R | | | | | CCAAATTTAGTCTACCTTGTGATC | | 88 | *Rhipicephalus sanguineus* | |  |
|  | | |  | | Ri_co_sca1_P | | | | | TCCTCCTGACGTATTAAAAGAAGCTGAAGCT | |  |  | |  |
|  | | |  | | Ri_pa_atl_sca2_F | | | | | GATAATGAAAAGACAGTAGGACG | |  |  | |  |
| *Rickettsia parkeri* | | | sca2 | | Ri_pa_atl_sca2_R | | | | | GATCTAAACGCCCTACTGTCT | | 85 | Two cultures | | (30) |
|  | | |  | | Ri_pa_atl_sca2_P | | | | | TTTCATTATCTAAACGCCCTACGGTCTTTTC | |  |  | |  |
| *Rickettsia africae* | | | sca1 | | Ri_af_sca1_F  Ri_af_sca1_R  Ri_af_sca1_P | | | | | GATACGACAAGTACCTCGCAG GGATTATATACTTTAGGTTCGTTAG CAGATAGGAACAGTAATTGTAACGGAACCAG | | 122 | Culture | | (30) |
| *Rickettsia felis* | | | orfB | | Ri_fel_orfB_F  Ri_fel_orfB_R  Ri_fel_orfB_P | | | | | ACCCTTTTCGTAACGCTTTGC TATACTTAATGCTGGGCTAAACC AGGGAAACCTGGACTCCATATTCAAAAGAG | | 163 | Culture | | (30) |
| *Rickettsia typhi* | | | ompB | | Ri_typ_ompB_F  Ri_typ_ompB_R  Ri_typ_ompB_P | | | | | CAGGTCATGGTATTACTGCTCA GCAGCAGTAAAGTCTATTGATCC ACAAGCTGCTACTACAAAAAGTGCTCAAAATG | | 133 | Two cultures | | (30) |
| *Rickettsia prowazekii* | | | gltA | | Ri_pro_gltA_F  Ri_pro_gltA_R  Ri_pro_gltA_P | | | | | CAAGTATCGGTAAAGATGTAATCG TATCCTCGATACCATAATATGCC ATATAAGTAGGGTATCTGCGGAAGCCGAT | | 151 | Plasmid | | (30) |
| *Borrelia*spp. | | | 23S rRNA | | Bo_bu_sl_23S_F  Bo_bu_sl-23S_R  Bo_bu_sl_23S_P | | | | | GAGTCTTAAAAGGGCGATTTAGT CTTCAGCCTGGCCATAAATAG TAGATGTGGTAGACCCGAAGCCGAGT | | 73 | ** Culture of *B. afzelii*,  *B. garinii*,  *B.valaisiana*, *B.lusitaniae*, *B. recurrentis* | | (32) |
| *Borrelia anserina* | | | Fla | | Bor_ans_fla_F  Bor_ans_fla_R  Bor_ans_fla_P | | | | | GGAGCACAACAAGAGGGAG TTGGAGAATTAACCCCACCTG TGCAAGCAACTCCAGCTCCAGTAGCT | | 76 | Plasmid | | (30) |
| *Borrelia lonestari* | | | glpQ | | Bor_lon_glpQ_F  Bor_lon_glpQ_R  Bor_lon_glpQ_P | | | | | GATCCAGAACTTGATACAACCAC TTCATCTAGTGAGAAGTCAGTAG AGTAATATCGTCCGTCTTCCCTAGCTCG | | 99 | Naturally infected *A. americanum* | | (30) |
| *Borrelia parkeri* | | | gyrB | | Bor_par_gyrB_F  Bor_par_gyrB_R  Bor_par_gyrB_P | | | | | GCAAAACGATTCAAAGTGAGTCC CTCATTGCCTTTAAGAAACCACTT TTAAAACCAGCAACATGAGTTCCTCCTTCTC | | 184 | 2 cultures | | (30) |
| *Borrelia bissettii* | | | rpoB | | Bo_bi_rpoB_F  Bo_bi_rpoB_R  Bo_bi_rpoB_P | | | | | GCAACCAGTCAGCTTTCACAG CAAATCCTGCCCTATCCCTTG AAAGTCCTCCCGGCCCAAGAGCATTAA | | 118 | Plasmid | | (32) |
| *Borrelia turicatae* | | | ITS | | Bor_tur_ITS_F  Bor_tur_ITS_R  Bor_tur_ITS_P | | | | | GACAAATGTTTATTGCATACGGC TAAACTCAACCCCTCTTAAGCAT AAGTTTGGTTGAAGCATCAGAAAGGCATCTTT | | 101 | 2 cultures | | (30) |
| *Borrelia burgdorferi* sensu stricto | | | glpA | | Bo_bu_glpA_F  Bo_bu_glpA_R  Bo_bu_glpA_P | | | | | GCAATTACAAGGGGGTATAAAGC GGCGTGATAAGTGCACATTCG TTAATTAAACGGGGTGCATTCTTCTCAAGAATG | | 206 | Culture | | (30) |
| *Borrelia theileri* | | | glpQ | | Bo_th_glpQ_F  Bo_th_glpQ_R  Bo_th_glpQ_P | | | | | GTGCTAACAAAGGACAATATTCC GGTTAGTGGAAAACGGTTAGGAT TATTATAATTCACGAGCCAGAGCTTGACAC | | 213 | Plasmid | | (30) |
| *Bartonella*spp. | | | ssrA | | Bart_spp_ssrA_F  Bart_spp_ssrA_RBart_spp_ssrA_P | | | | | CGTTATCGGGCTAAATGAGTAG ACCCCGCTTAAACCTGCGA TTGCAAATGACAACTATGCGGAAGCACGTC | | 118 | **, Culture of *B. quintana* | | (30) |
| *Bartonella barcilliformis* | | | rpoB | | Ba_ba_rpoB_F  Ba_ba_rpoB_R  Ba_ba_rpoB_P | | | | | GAAGAGTTTGTAGTTTGTCGTCA AGCAGCTACAGAAACCAACTG TGCAGGTGAAGTTTTGATGGTGCCACG | | 105 | Culture | | (32) |
| *Bartonella henselae* | | | ribC | | Bar_he_ribC_F  Bar_he_ribC_R Bar_he_ribC_P | | | | | GGGATGCGATTTAATAGTTCTAC CGCTTGTTGTTTTGATCCTCG ACGTTATAGTAGCGAAAACTTAGAAATTGGTGC | | 116 | Three cultures | | (30) |
| *Bartonella vinsonii*subsp.  *Berkhoffii* | | | ITS | | Bar_vin_ITS_F  Bar_vin_ITS_R  Bar_vin_ITS_P2 | | | | | GGAATTGCTTAACCCACTGTTG CCTTATTGATTTAGATCTGATGGG AGAAACTCCCGCCTTTATGAGAGAAATCTCT | | 141 | Four cultures | | (30) |
| *Coxiella burnetii* | | | Icd | | Co_bu_icd_F  Co_bu_icd_R  Co_bu_icd_P | | | | | AGGCCCGTCCGTTATTTTACG CGGAAAATCACCATATTCACCTT TTCAGGCGTTTTGACCGGGCTTGGC | | 74 | Culture | | (32) |
| *Coxiella*- like endosymbiont | | | IS1111 | | | | Co_bu_IS111_F  Co_bu_IS111_R  Co_bu_IS111_P | | | TGGAGGAGCGAACCATTGGT CATACGGTTTGACGTGCTGC ATCGGACGTTTATGGGGATGGGTATCC | | 86 | Culture | | (32) |
| *Francisella tularensis* | | | tul4 | | | | Fr_tu_tul4_F  Fr_tu_tul4_R  Fr_tu_tul4_P | | | ACCCACAAGGAAGTGTAAGATTA GTAATTGGGAAGCTTGTATCATG AATGGCAGGCTCCAGAAGGTTCTAAGT | | 76 | Culture | | (32) |
| *Francisella*-like  endosymbiont | | | fopA | | | | Fr_tu_fopA_F  Fr_tu_fopA_R  Fr_tu_fopA_P | | | GGCAAATCTAGCAGGTCAAGC CAACACTTGCTTGAACATTTCTAG AACAGGTGCTTGGGATGTGGGTGGTG | | 91 | Culture | | (32) |
| *Anaplasma* spp. | | | | 16S rRNA | | Ana_spp_16S_F  Ana_spp_16S_R  Ana_spp_16S_P | | | | CTTAGGGTTGTAAAACTCTTTCAG  CTTTAACTTACCAAACCGCCTAC  ATGCCCTTTACGCCCAATAATTCCGAACA | | 160 | | ** | (30) |
| *Anaplasma marginale* | | | | *msp1b* | | An_ma_msp1_F  An_ma_msp1_F  An_ma_msp1_R | | | | CAGGCTTCAAGCGTACAGTG  GATATCTGTGCCTGGCCTTC  ATGAAAGCCTGGAGATGTTAGACCGAG | | 85 | | Experimentally infected bovine blood sample | (32) |
| *Anaplasma phagocytophilum* | | | | msp2 | | An_ph_msp2_F An_ph_msp2_R  An_ph_msp2_P | | | | GCTATGGAAGGCAGTGTTGG GTCTTGAAGCGCTCGTAACC AATCTCAAGCTCAACCCTGGCACCAC | | 77 | | Infected *Ixodes* spp. tick | (32) |
| *Anaplasma platys* | | | | groEL | | | An_pla_groEL_F  An_pla_groEL_R  An_pla_groEL_P | | | TTCTGCCGATCCTTGAAAACG  CTTCTCCTTCTACATCCTCAG  TTGCTAGATCCGGCAGGCCTCTGC | | 75 | | Infected canine blood sample | (32) |
| *Anaplasma bovis* | | | | groEL | | | An_bo_groEL_F  An_bo_groEL_R  An_bo_groEL_P | | | GGGAGATAGTACACATCCTTG CTGATAGCTACAGTTAAGCCC AGGTGCTGTTGGATGTACTGCTGGACC | | 73 | | Plasmid | (32) |
| *Anaplasma ovis* | | | | msp4 | | | An_ov_msp4_F An_ov_msp4_r An_ov_msp4_P | | | TCATTCGACATGCGTGAGTCA TTTGCTGGCGCACTCACATC AGCAGAGAGACCTCGTATGTTAGAGGC | | 92 | | Plasmid | (32) |
| *Ehrlichia* spp. | | | | 16S rRNA | | | Neo_mik_16S_F  Neo_mik_16S_R  Neo_mik_16S_P | | | GCAACGCGAAAAACCTTACCA  AGCCATGCAGCACCTGTGT  AAGGTCCAGCCAAACTGACTCTTCCG | | 98 | | ** | (32) |
| *Ehrlichia canis* | | | | gltA | | | Eh_ca_gltA_F  Eh_ca_gltA_R  Eh_ca_gltA_P | | | GACCAAGCAGTTGATAAAGATGG CACTATAAGACAATCCATGATTAGG ATTAAAACATCCTAAGATAGCAGTGGCTAAGG | | 136 | | Culture | (30) |
| *Ehrlichia chaffeensis* | | | | dsb | | | Eh_ch_dsb_F  Eh_ch_dsb_R  Eh_ch_dsb_P | | | TATTGCTAATTACCCTCAAAAAGTC  GAGCTATCCTCAAGTTCAGATTT  ATTGACCTCCTAACTAGAGGGCAAGCA | | 117 | | Infected *Amblyomma americanum* | (32) |
| *Ehrlichia ewingii* | | | | dsb | | | Eh_ew_dsb_F  Eh_ew_dsb_R  Eh_ew_dsb_P | | | CAATACTTGGAGAAGCATCATTG  TTGCTTATGGCTTAATGCTGCAT  AAAGCAGTACGTGCAGCATTGGCTGTA | | 111 | | Infected *Amblyomma americanum* | (32) |
| *Ehrlichia ruminantium* | | | | gltA | | | Eh_ru_gltA_F  Eh_ru_gltA_R  Eh_ru_gltA_P | | | CCAGAAAACTGATGGTGAGTTAG  AGCCTACATCAGCTTGAATGAAG  AGTGTAAACTTGCTGTTGCTAAGGTAGCATG | | 116 | | Culture | (30) |
| Panola Mountain *Ehrlichia* | | | | gltA | | | Eh_PME_gltA_F  Eh_PME_gltA_R  Eh_PME_gltA_P | | | GCTAGTTATGAGTTAGAATGTAAAC TACTATAGGATAATCTTGAATCAGC TTGCTATCGCTAAAATTCCAAGTATGATTGCG | | 121 | | Infected *Amblyomma americanum* | (30) |
| *Neoehrlichia mikurensis* | | | | groEL | | | Neo_mik_groEL_F  Neo_mik_groEL_R  Neo_mik_groEL_P | | | AGAGACATCATTCGCATTTTGGA TTCCGGTGTACCATAAGGCTT AGATGCTGTTGGATGTACTGCTGGACC | | 96 | | Infected rodent sample | (32) |
| *Aegyptianella pullorum* | | | | groEL | | | Ae_pul_groEL_F  Ae_pul_groEL_R  Ae_pul_groEL_P | | | AGCCAGTATTATCGCTCAAGG  GCCTCACGTGCCTTCATAAC  TGCTTCTCAGTGTAACGACAGGGTTGG | | 168 | | Plasmid | (30) |
| Apicomplexa | | | | 18S rRNA | | | Apic_18S_F  Apic_18S_R  Apic_18S_P | | | TGAACGAGGAATGCCTAGTATG  CACCGGATCACTCGATCGG  TAGGAGCGGGCGGTGTGTAC | | 104 | | anine blood with *B. canis,*  *B. rossi*, culture of  *B. divergens, T. lestoquari* and  *T. annulata* | (30) |
| *Babesia vogeli* | | | | Hsp70 | | | | | Ba_vo_hsp70_F  Ba_vo_hsp70_R  Ba_vo_hsp70_P | TCACTGTGCCTGCGTACTTC  TGATACGCATGACGTTGAGAC  AACGACTCCCAGCGCCAGGCCAC | | 87 | | Infected canine blood sample | (32) |
| *Babesia ovis* | | | | 18S rRNA | | | | | Ba_ov_18S_F  Ba_ov_18S_R  Ba_ov_18S_P | TCTGTGATGCCCTTAGATGTC  GCTGGTTACCCGCGCCTT  TCGGAGCGGGGTCAACTCGATGCAT | | 92 | | Plasmid | (32) |
| *Babesia bigemina* | | | | 18S rRNA | | | | | Ba_big_RNA18S_F  Ba_big_RNA18S_R  Ba_big_RNA18S_P | ATTCCGTTAACGAACGAGACC TTCCCCCACGCTTGAAGCA  CAGGAGTCCCTCTAAGAAGCAAACGAG | | 99 | | Plasmid | (32) |
| *Babesia gibsoni* | | | | Rap1 | | | | | Ba_gib_rap1_F  Ba_gib_rap1_R  Ba_gib_rap1_P | CTCTTGCTCATCATCTTTTCGG  TCAGCGTATCCATCCATTATATG  TTTAATGCGTGCTACGTTGTACTTCCCAAAG | | 130 | | Plasmid | (30) |
| *Babesia caballi* | | | | Rap1 | | | | | Ba_cab_rap1_F  Ba_cab_rap1_R  Ba_cab_rap1_P | GTTGTTCGGCTGGGGCATC  CAGGCGACTGACGCTGTGT  TCTGTCCCGATGTCAAGGGGCAGGT | | 94 | | Plasmid | (32) |
| *Babesia bovis* | | | | CCTeta | | | | | Ba_bo_CCTeta_F  Ba_bo_CCTeta_R  Ba_bo_CCTeta_P | GCCAAGTAGTGGTAGACTGTA  GCTCCGTCATTGGTTATGGTA  TAAAGACAACACTGGGTCCGCGTGG | | 100 | | Plasmid | (32) |
| *Babesia duncani* | | | | ITS2 | | | | | Ba_du_ITS_F  Ba_du_ITS_R  Ba_du_ITS_P | ATTTCCGTTTGCGAGAGTTGC AGGAAGCATCAAGTCATAACAAC  AACAAGAGGCCCCGAGATCAAGGCAA | | 87 | | Plasmid | (32) |
| *Babesia microti* | | | | CCTeta | | | | | Bab_mi_CCTeta_F  Bab_mi_CCTeta_R  Bab_mi_CCTeta_P | ACAATGGATTTTCCCCAGCAAAA  GCGACATTTCGGCAACTTATATA  TACTCTGGTGCAATGAGCGTATGGGTA | | 145 | | Culture | (32) |
| *Theileria parva* | | | | 18S rRNA | | | | | Th_pa_18S_F  Th_pa_18S_R  Th_pa_18S_P | GAGTATCAATTGGAGGGCAAG  CAGACAAAGCGAACTCCGTC  AAATAAGCCACATGCAGAGACCCCGAA | | 173 | | Culture | (32) |
| *Theileria mutans* | | | | ITS | | | | | The_mu_ITS_F  The_mu_ITS_R  The_mu_ITS_P | CCTTATTAGGGGCTACCGTG  GTTTCAAATTTGAAGTAACCAAGTG  ATCCGTGAAAAACGTGCCAAACTGGTTAC | | 119 | | Plasmid | (30) |
| *Theileria velifera* | | | | 18S rRNA | | | | | The_ve_18S_F  The_ve_18S_R  The_ve_18S_P | TGTGGCTTATCTGGGTTCGC CCATTACTTTGGTACCTAAAACC  TTGCGTTCCCGGTGTTTTACTTTGAGAAAG | | 151 | | Plasmid | (30) |
| *Theileria equi* | | ema1 | | | | | | | Th_eq_ema1_F4  Th_eq_ema1_R4  Th_eq_ema1_P4 | CGGCAAGAAGCACACCTTC  TGCCATCGCCCTTGTAGAG  AAGGCTCCAGGCAAGCGCGTCCT | | 167 | | Plasmid | (30) |
| *Cytauxzoon felis* | ITS2 | | | | | | Cy_fel_ITS2_F  Cy_fel_ITS2_R  Cy_fel_ITS2_S | | AAGATCCGAACGGAGTGAGG  GTAGTCTCACCCAATTTCAGG AAGTGTGGGATGTACCGACGTGTGAG | | 119 | | Plasmid | (30) |  |
|  |  | | | | | | Hepa_spp_18SF | | ATTGGCTTACCGTGGCAGTG | |  | |  | (30) |  |
| *Hepatozoon*spp. | 18S | | | | | | Hepa_spp_18S_R | | AAAGCATTTTAACTGCCTTGTATTG | | 175 | | ** |  |  |
|  |  | | | | | | Hepa_spp_18S_S | | ACGGTTAACGGGGGATTAGGGTTCGAT | |  | |  |  |  |
| *Hepatozoon canis* | 18S rRNA | | | | | | He_can_18S_F | | TTCTAACAGTTTGAGAGAGGTAG | |  | |  | (30) |  |
|  |  | | | | | | He_can_18S_R | | AGCAGACCGGTTACTTTTAGC | | 221 | | Dog blood |  |  |
|  |  | | | | | | He_can_18S_S | | AGAACTTCAACTACGAGCTTTTTAACTGCAAC | |  | |  |  |  |
| *Hepatozoon americanum* | 18S rRNA | | | | | | He_ame_18S_F2  He_ame_18S_R2  He_ame_18S_P2 | | GGTATCATTTTGGTGTGTTTTTAAC  CTTATTATTCCATGCTCCAGTATTC  AAAAGCGTAAAAGCCTGCTAAAAACACTCTAC | | 159 | | Plasmid | (30) |  |
| *Leishmania*spp. | hsp70 | | | | | | Leish_spp_hsp70_F  Leish_spp_hsp70_R Leish_spp_hsp70_S | | CGACCTGTTCCGCAGCAC  TCGTGCACGGAGCGCTTG  TCCATCTTCGCGTCCTGCAGCACG | | 78 | | Culture of  *L. martini-quensis* | (30) |  |
|  |  | | | | | | Le_inf_ITS_F | | CGCACCGCCTATACAAAAGC | |  | | Culture | (30) |  |
| *Leishmania* | ITS | | | | | | Le_inf_ITS_R | | GTTATGTGAGCCGTTATCCAC | | 103 | |  |  |  |
| *infantum* |  | | | | | | Le_inf_ITS_S | | ACACGCACCCACCCCGCCAAAAAC | |  | |  |  |  |
| *Rangelia vitalii* | 18S rRNA | | | | | | Ra_vit_18S_F  Ra_vit_18S_R  Ra_vit_18S_S | | TAACCGTGCTAATTGTAGGGC  GAATCACCAAACCAAATGGAGG  TAATACACGTTCGAGGGCGCGTTTTGC | | 92 | | Plasmid | (30) |  |
|  |  | | | | | | Tick_spp_16S_F | | AAATACTCTAGGGATAACAGCGT | |  | |  |  |  |
| *Tick*spp. | 16S rRNA | | | | | | Tick_spp_16S_R | | TCTTCATCAAACAAGTATCCTAATC | | 99 | | ** | (30) |  |
|  |  | | | | | | Tick_spp_16S_P | | CAACATCGAGGTCGCAAACCATTTTGTCTA | |  | |  |  |  |
| *Amblyomma*  *variegatum* | ITS2 | | | | | | Amb_var_ITS2_F  Amb_var_ITS2_R  Amb_var_ITS2_P | | GCCAGCCTCTGAAGTGACG  TTCTGCGGTTTAAGCGACGC  TCTTGCCACTCGACCCGTGCCTC | | 117 | | Tick extract, Guadeloupe | (30) |  |
| *Rhipicephalus microplus* | ITS2 | | | | | | Rhi_mic_ITS2_F  Rhi_mic_ITS2_R  Rhi_mic_ITS2_P | | GCTTAAGGCGTTCTCGTCG  CAAGGGCAGCCACGCAG  TAGTCCGCCGTCGGTCTAAGTGCTTC | | 144 | | Tick extract, Galapagos Islands | (30) |  |
| *Rhipicephalus sanguineus*  sensu lato | ITS2 | | | | | | Rhi_san_ITS2_F  Rhi_san_ITS2_R  Rhi_san_ITS2_P | | TTGAACGCTACGGCAAAGCG  CCATCACCTCGGTGCAGTC  ACAAGGGCCGCTCGAAAGGCGAGA | | 110 | | Tick extract, France | (30) |  |
| *Mycoplasma* spp. | 16S rRNA | | | | | | Myc_spp_16S_F  Myc_spp_16S_R  Myc_spp_16S_P | | GTGACGGCTAACTATGTGCC  GCTTTACGCCCAATAATTCCG  AGCAGCTGCGGTAATACATAGGTCGC | | 77 | | Culture | (30) |  |
| *Mycoplasma ovis* | rnpB | | | | | | Myc_ov_rnpB_F  Myc_ov_rnpB_R  Myc_ov_rnpB_P | | GACTCAACGACTAGTTCTACTAG CTATAAAGCTCGTCTCTGTGG TTGATGGCGGAGAACTTTTCTAAATAAGCC | | 106 | | Plasmid | (30) |  |
| *Mycoplasma haemocanis* | 23S rRNA | | | | | | Myc_hca_23S_F  Myc_hca_23S_R  Myc_hca_23S_P | | AGCGGGTTATTGGATTCTCGT  AGAATATGATGTTTGCGACTTCG  TTAAGCTGCAAGTGTTTCGGGTAGTAAAATAC | | 126 | | Plasmid | (30) |  |
| *Candidatus Mycoplasma haematoparvum* | 16S rRNA | | | | | | Myc_hpa_16S_F  Myc_hpa_16S_R  Myc_hpa_16S_P | | CGAAGAGGGCTTGCCCTC  CCTTTCGGATTGCTATTCTCC  TTAGTGGCGAACGGGCGAGTAACGC | | 85 | | Plasmid | (30) |  |
| *Mycoplasma suis* | rnpB | | | | | | Myc_su_rnpB_F  Myc_su_rnpB_R  Myc_su_rnpB_P | | ACTTAACGGCTAGAGAAATCTAG  TTTACCGCGTTTCACACTTTTAC  ATTTCTGTAAAGCTCGTCTCTGTGGCACT | | 131 | | Plasmid | (30) |  |
| *Mycoplasma haemofelis* | dnaK | | | | | | Myc_hfe_dnaK_F  Myc_hfe_dnaK_R  Myc_hfe_dnaK_P | | TTGGCTACTTCTGGTGATAACC  GTGTTCCTTCTTGATTTCCTCTA  ATTTGGGTGGTGACGATTGGGATCAAGC | | 90 | | Plasmid | (30) |  |
| *Candidatus Mycoplasma haemominutum* | 16S rRNA | | | | | | Myc_hmi_16S_F  Myc_hmi_16S_R  Myc_hmi_16S_P | | CTCGCGAGCAGAGAGGAG  ATATGGGCCGTATTTCAATCCC  ATTGCGGCTGGTCGACCCCTCAGT | | 123 | | Plasmid | (30) |  |
| *Mycoplasma turicensis* | rnpB | | | | | | Myc_htu_rnpB_F  Myc_htu_rnpB_R  Myc_htu_rnpB_P | | GACGGCTGTAGTGATGATGC  GATTCTGAAATAGTAACTCTC  TTAGTTTCAGAATCGCCATCACCTTTTGGG | | 100 | | Plasmid | (30) |  |
| *Mycoplasma wenyonii* | 23S rRNA | | | | | | Myc_we_23S_F  Myc_we_23S_R  Myc_we_23S_P | | CACAAGAGTGAGAATCGAATACG  TATTCCTAGAGTCTTTTCTGGAG  AACGTGATTAAGTCTGGCTCTCTCAGC | | 89 | | Plasmid | (30) |  |
| *Escherichia coli* | Eae | | | | | | eae-F  eae-R  eae-P | | CATTGATCAGGATTTTTCTGGTGATA  CTCATGCGGAAATAGCCGTTA  ATAGTCTCGCCAGTATTCGCCACCAATACC | | 102 | | Culture of EDL933 strain | (32) |  |

**Includes all controls belonging to the genus described in the table and targeted by a specific design.

**Supplementary Table 2.** Frequency distribution of pathogens found in dual infected ticks removed from dogs*.

|  | ***Rhipicephalus sanguineus* s.l.******  **(N=481)** | | | ***Amblyomma variegatum***  **(N=8)** | **No. (%)**  **N=505** |
| --- | --- | --- | --- | --- | --- |
| **Pathogens** | **Barbados**  **(n=87)** | **St. Kitts**  **(n=119)** | **Trinidad**  **(n=238)** | **Barbados**  **(n=2)** |  |
| ***Anaplasma marginale/Babesia vogeli*** | 0 | 0 | 1 (0.4) | 0 | 1 (0.2) |
| ***Anaplasma marginale/Babesia bovis*** | 0 | 0 | 1 (0.4) | 0 | 1 (0.2) |
| ***Anaplasma platys/Rickettsia* spp.** | 0 | 1 (0.8) | 0 | 0 | 1 (0.2) |
| ***Borrelia* spp.*/Theileria mutans*** | 0 | 1 (0.8) | 0 | 0 | 1 (0.2) |
| ***Ehrlichia canis/Hepatozoon canis*** | 0 | 0 | 1 (0.4) | 0 | 1 (0.2) |
| ***Ehrlichia ruminantium/Rickettsia africae*** | 1 (1.1) | 0 | 0 | 1 (50) | 2 (0.4) |
| **Total no. of dual-infected ticks** | **1 (1.1)** | **2 (1.7)** | **3 (1.3)** | **1 (50)** | **7 (1.4)** |

*Territories with no detection of tick pathogens have been omitted.

**Supplementary Table 3:** Frequency distribution of pathogens found in ticks removed from dogs with triple infections*.

|  | ***Rhipicephalus sanguineus* s.l. (%)**  **(N=481)** | ***Amblyomma ovale* (%)**  **(N=21)** | ***Amblyomma variegatum* (%)**  **(N=8)** | **No. (%)** |
| --- | --- | --- | --- | --- |
|  | **Trinidad**  **(n=238)** | **Trinidad**  **(n=238)** | **Barbados**  **(n=2)** |  |
| ***A. marginale/E. canis/Ehrlichia* spp.** | 0 | 1 (5.0) | 0 | 1 (0.1) |
| ***E. ruminantium/R. africae/R. parkeri*** | 0 | 0 | 1 (50) | 1 (0.1) |
| ***H. canis/E. canis/Ehrlichia* spp.** | 1 (0.4) | 0 | 0 | 1 (0.1) |
| **Total no. of triple-infected ticks** | **1 (0.4)** | **1 (5)** | **1 (50)** | **3 (0.6)** |

*Territories with no detection of tick pathogens have been omitted

**Supplementary Table 4.** Frequency distribution of pathogens found in dual-infected ticks removed from cattle*.

|  | ***Rhipicephalus microplus***  **(N=326)** | | | | ***Amblyomma variegatum***  **(N=8)** | ***Amblyomma cajennense***  **(N=4)** | **No. (%)**  **N=335** |
| --- | --- | --- | --- | --- | --- | --- | --- |
|  | **Barbados**  **(n=37)** | **St. Kitts**  **(n=41)** | **St. Lucia**  **(n=97)** | **Trinidad**  **(n=91)** | **Barbados**  **(n=2)** | **Guyana**  **(n=4)** |  |
| ***A.marginale/Ehrlichia* spp.** | 4 (10.8) | 1 (2.4) | 0 | 4 (4.4) | 0 | 0 | 9 (1.1) |
| ***Anaplasma marginale/Rickettsia* spp.** | 0 | 2 (4.9) | 0 | 1 (1.1) | 0 | 0 | 3 (0.4) |
| ***Anaplasma marginale/Rickettsia africae*** | 0 | 0 | 2 (2.1) | 0 | 0 | 0 | 2 (0.2) |
| ***Anaplasma marginale/Babesia vogeli*** | 0 | 0 | 0 | 1 (1.1) | 0 | 1 (25.0) | 2 (0.2) |
| ***Anaplasma marginale/Rickettsia felis*** | 0 | 0 | 0 | 1 (1.1) | 0 | 0 | 1 (0.1) |
| ***Ehrlichia* spp.*/Rickettsia* spp.** | 0 | 0 | 1 (1.0) | 0 | 0 | 0 | 1 (0.1) |
| ***Ehrlichia* spp*./Mycoplasma wenyonii*** | 0 | 0 | 0 | 1 (1.1) | 0 | 0 | 1 (0.1) |
| ***Ehrlichia* spp.*/Theileria mutans*** | 0 | 1 (2.4) | 0 | 0 | 0 | 0 | 1 (0.1) |
| ***Ehrlichia ruminantium/Rickettsia africae*** | 0 | 0 | 0 | 0 | 1 (50) | 0 | 1 (0.1) |
| **Total no. of dual infected ticks** | **4 (10.8)** | **4 (9.8)** | **3 (3.1)** | **8 (8.8)** | **1 (50)** | **1 (25)** | **21 (6.3)** |

*Territories with no detection of tick pathogens have been omitted

**Supplementary Table 5:** Frequency distribution of pathogens found in ticks removed from cattle with triple infections.

|  | ***Rhipicephalus microplus (%)***  **(N=326)** | | **No. (%)**  **N=335** |
| --- | --- | --- | --- |
|  | **St. Kitts**  **(n=41)** | **St. Lucia**  **(n=97)** |  |
| ***A.marginale/Ehrlichia spp./Borrelia* spp.** | 0 | 1 (1) | 1 (0.1) |
| ***A.marginale/B.bigemina/R. africae*** | 1 (2.4) | 0 | 1 (0.1) |
| **Total no. of triple-infected ticks** | **1 (2.4)** | **1 (1)** | **2 (0.6)** |

*Territories with no detection of tick pathogens have been omitted
